# Wood and Cellulose: the Most Sustainable Advanced Materials for Past, Present, and Future Civilizations

**DOI:** 10.1002/adma.202415787

**Published:** 2025-01-07

**Authors:** Mahiar Max Hamedi, Mats Sandberg, Richard T. Olsson, Jan Pedersen, Tobias Benselfelt, Jakob Wohlert

**Affiliations:** ^1^ Department of Fiber and Polymer Technology School of Engineering Sciences in Chemistry Biotechnology and Health KTH Royal Institute of Technology Stockholm 10044 Sweden; ^2^ Wallenberg Wood Science Center KTH Royal Institute of Technology Stockholm 10044 Sweden; ^3^ RISE Research Institutes of Sweden AB Digital Systems Smart Hardware Printed, Bio‐ and Organic Electronics Södra Grytsgatan 4 Norrköping 60233 Sweden; ^4^ NCAB Group AB Löfströms allé 5 Sundbyberg 17266 Sweden

**Keywords:** advanced sustainable materials, cellulose, energy, electronics, wood

## Abstract

Wood, with its constituent building block cellulose, is by far the most common biomaterial on the planet and has been the most important material used by humans to establish civilization. If there is one single biomaterial that should be studied and used by materials scientists across disciplines to achieve a sustainable future, it is cellulose. This perspective provides insights for the general materials science community about the unique properties of wood and cellulose and how they may be used in advanced sustainable materials to make a substantial societal impact. The focus is on sawn wood or cellulose fibers produced at scale by industry and the more recent cellulosic nanomaterials, highlighting the areas where these cellulose‐based materials can be valorized into higher‐order functions. Numerous articles have comprehensively reviewed different areas where cellulose is currently used in advanced materials science. The objective here is to provide general insight for all material scientists and to provide the opinions about the areas in which cellulose and wood have the largest potential to make a significant societal impact, especially to realize next‐generation sustainable materials for construction, food, water, energy, and information. Discussing key areas where future research is needed to open avenues toward a more sustainable future is ended.

## Graphical abstract

Wood and cellulose are the most abundant and important sustainable materials on the planet at the disposal to solve major societal challenges. This perspective, written for all materials scientists, highlights how breakthroughs in cellulose nanotechnology combined with functional nanomaterials can revolutionize important areas like construction, electronics, recycling, and intelligent materials at scale, toward a sustainable future.

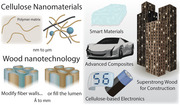

## 1 Wood and Cellulose Civilized Man

Terrestrial plants are by far the dominant kingdom on planet Earth,^[^ ^1^ ^]^ containing ≈450 gigatons of carbon (a measure of biomass), compared to animals (2 gigatons of carbon) and bacteria/archaea (80 gigatons of carbon). As such, cellulose, which originated in the biosynthesis of cyanobacteria for over 3.5 billion years^[^ ^2^ ^]^ and has migrated into the plant kingdom in eons, is currently the main building block of life on Earth and the most abundant, well‐defined natural polymer at our disposal.^[^ ^3^ ^]^

Lieth & Whittaker calculated in 1975 that the standing crop on Earth produced ≈2 × 10^11^ tons of material per year, of which half is cellulose. The global standing crop, therefore, contains ≈10^11^ tons of cellulose. Considering that life has a substantial biochemical diversity, it is astonishing that one well‐defined compound, cellulose, contributes so much to Earth's entire biomass.^[^ ^4^ ^]^ For that reason, it is not surprising that wood has been the primary material that civilized man. Initially, our ancestorial species used it to make fire, as far back as the lower Pleistocene, ≈2 million years ago.^[^ ^5^ ^]^ Wood has, therefore, even accelerated the evolution of our species, as some fire‐making hominids spent an order of magnitude less time feeding. This is suggested to have led to a significant change in the rate of evolution, catalyzing the split between chimpanzees and humans.

**Figure 1:**
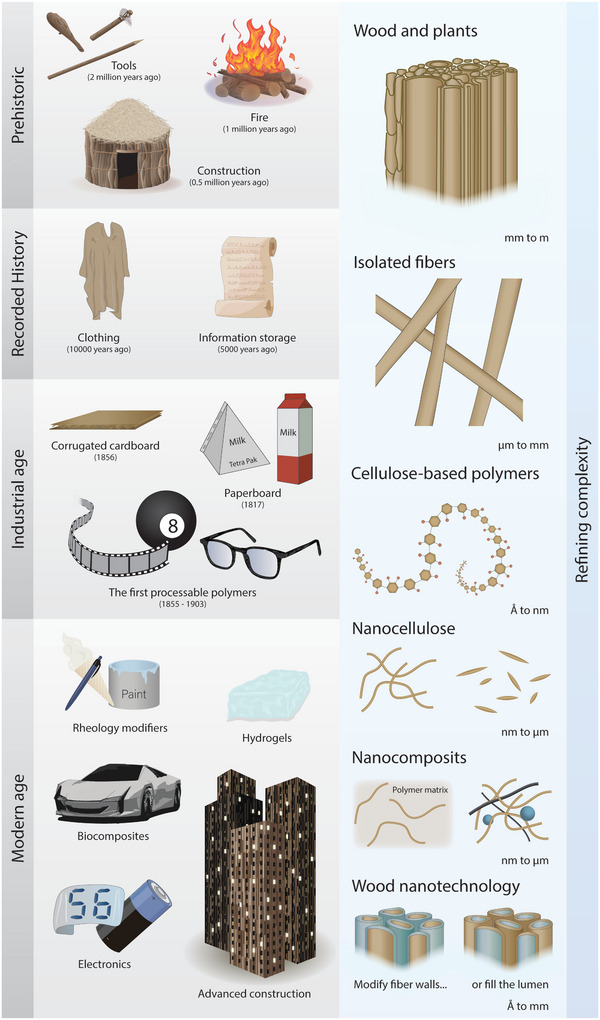
Schematic representation of wood and cellulose‐based materials and their past, present, and future development cycle and impact on human civilizations.

In ancient times, the making of tools and building of houses with wood enabled settlements and cities. Later, wood enabled us to revolutionize the spread of information with paper, and in the last century, cellulose opened the path for modern polymer chemistry and synthetic plastics (**Figure** **1**).

Industrial processing of pulp in the 1900s led to the scale‐up of the wood and paper industry, which today provides us with a large amount of renewable raw materials. The production of pulp for paper and board is ≈400 million tonnes in 2020, while sawn wood, panels, and other wood products collectively accounted for ≈1 billion tonnes per year.^[^ ^6^ ^]^ This currently established wood/pulp industry has also become increasingly more sustainable with better management of tree plantations. Indeed, the growth of forests for producing pulp and paper is greater than the amount of felled trees and has been going on for the entire 20th century onward in countries like Sweden.^[^ ^7^ ^]^

Wood‐based materials are already the most important, if not the only, basic human production flow at scale, which can constantly feed our societies with advanced materials. If there is one single biomaterial that should be studied and used by materials scientists across disciplines toward a sustainable future, it should be wood and cellulose. To dig deeper, we must understand cellulose's role in creating the polymer age and its current role in the nano‐age.

## 2 Cellulose Enabled the Polymer Age

Even though wood and paper enabled modern civilization, they were first described systematically by Anselme Payen. Payen showed that the fibrous structure in plant tissues could withstand organic and aqueous solvents and be extracted. Payen then determined that the extracted material had a molecular composition resembling starch, with the formula (C₆H₁₀O₅)_n_, and named it “cellulose” in 1838.^[^ ^8^ ^]^

It would take another century, however, until the polymer structure of cellulose was revealed in 1920 by the ground‐breaking work of Hermann Staudinger.^[^ ^9^ ^]^ Staudinger used acetylation and de‐acetylation of extracted cellulose to prove that the glucose units of this material were linked to each other covalently, forming very long molecular chains. In essence, this work marks the starting point for polymer science.^[^ ^3^ ^]^

Indeed, the extraction of polymers from wood occurred before Staudinger's work, predating the establishment of polymer theories. This already enabled several industrial products, like rayon/cellophane, which were the first “man‐made” sheets and fibers, and cellulose acetate, which enabled revolutionary products like reading glass frames, the scotch tape developed by 3 M, and modern photographic films democratizing photography by Kodak.^[^ ^10^ ^]^

**Figure 2:**
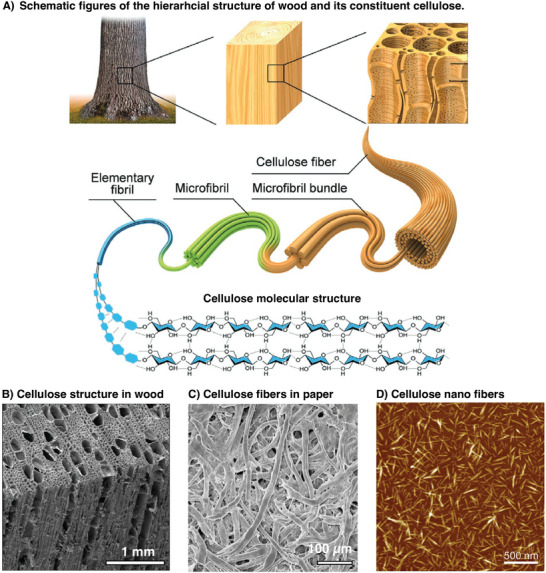
A) Schematic representation of the hierarchical structure of wood. Reproduced from Ref. [12] with permission from Wiley. B) SEM image of wood. Reproduced with permission from Technische Universität Dresden, Germany. C) SEM image of cellulose paper, D) AFM image of cellulose nanofibers/crystals. Reproduced from Ref. [13] with permission from Elsevier.

Cellulose is a linear polymer of glucose molecules (**Figure** **2**A) in which individual glucose units are connected via acetal linkages between the C1 and C4 carbons of the glucopyranose rings (Figure 2). The cellulose polymer can reach extreme lengths up to 15 000 glucose units, which is indeed even today longer than most synthetic polymers, only surpassed by a few materials like ultra‐high molecular weight polyethylene. Every glucose monomer has equatorial hydroxyl groups and axial hydrogen atoms perpendicular to the face of the pyranose (saccharide) ring, and cellulose shows distinct hydrophilic and hydrophobic characteristics. Consequently, the polymer self‐associates and becomes insoluble in water. Aggregation is driven by the entropy change in water and non‐covalent interactions like van der Waals between the glucopyranose rings.^[^ ^11^ ^]^ The molecular structure of cellulose makes it hydrophilic, chiral, and degradable while allowing a large set of chemical tailoring through its reactive OH groups.

These groups also form large hydrogen bond networks, which give the cellulose a well‐defined hierarchical order in supramolecular structure and organization. The lowest form of structure in this hierarchy is nanofiber‐shaped structures in plant cell walls. The discovery of this first ordered cellulose nanofiber took wood into the nano‐age, as will be described in the next section.

## 3 Cellulose Enters the Nano‐Age

The journey that initiated the cellulose nano‐age started with the X‐ray investigations in 1920, showing crystalline structures in cellulose fibers. Still, it took another thirty years until Bengt Rånby isolated these crystalline nanocellulose structures in 1949 using acid hydrolysis.^[^ ^14^ ^]^ Today, they are known as cellulose nanocrystals CNCs (Figure 2D), which are, in turn, fragments of longer nanowires called cellulose nanofiber/fibrils (CNFs). Another milestone was the grinding of pulp into microfibrils, aggregates of nanofibrils, by Turbak et al. In 1983.^[^ ^15^ ^]^ However, since nanoscience and its potential were largely unknown in the 80s, the interest in cellulose nanoparticles re‐ignited in the 1990s with the discovery of the chiral nematic organization of cellulose nanocrystals.^[^ ^16^ ^]^ The latest breakthrough came with the realization of cellulose nanofibril dispersions introduced in the 2000s.^[^ ^17^, ^18^ ^]^ This was achieved by covalent functionalization of the cellulose surface of pulp fibers, for example, using TEMPO‐mediated oxidation chemistry that introduces carboxylate groups carrying a negative charge, allowing for further disintegration of the nanocellulose into colloidally stable dispersions. This discovery thus led to the mass production of high‐quality bio‐based nanofiber and enabled vibrant research fields in modern nanomaterials.

## 4 Cellulose Bottom‐Up and Top‐Down Nanotechnology

**Figure 3:**
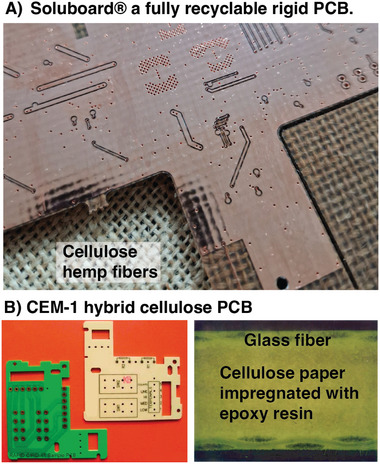
A) Photo of a fully recyclable rigid PCB “Soluboard” having a woven cellulose fiber core. Permission from JIVA materials. B) Photo and cross‐sectional image of CEM‐1 hybrid PCB board with a cellulose core, with permission from Ansys.

Nanocellulose has a chiral nematic ordering, which can be used as a tool to create advanced nanostructured materials with structural colors. However, the most exciting features of different nanocelluloses are their stiffness, reaching 140–150 GPa,^[^ ^19^ ^]^ their high aspect ratio of up to 1000 that allows them to form strong networks at low concentration, and their ability to disperse other nanoparticles in water for bottom‐up self‐assembly into superstructural nanocomposites.^[^ ^20^ ^]^ Some of the basic properties that have been measured for single nanofibers should define the theoretical limit for what could ultimately be achieved in their homogeneous nanocomposites, and we are far from having reached the full potential of these materials. For example, nanocellulose has been used to fabricate the strongest known synthetic bio‐based fibers with a tensile strength of up to 1.57 GPa and tensile stiffness of up to 86 GPa (**Figure** **3**).^[^ ^21^ ^]^ Nanocellulose has further been used to make solid, self‐supporting hydrogels containing as much as 99.99% water, which is amazing considering that seawater has 350 times less water per volume than these freestanding hydrogels.^[^ ^22^ ^]^ Nanocellulose can also form highly porous foams or aerogels with high surface area, low thermal expansion, and very light weight of down to 2 kg m^−3^.^[^ ^23^ ^]^ (see **Table** **1**). The colloidal stability of nanocellulose naturally allows them to be mixed with other water‐dispersed nanomaterials, especially synthetic electroactive materials like conducting polymers and 2D materials (e.g., graphene, MXene, TMDs). Indeed, the amphiphilic feature of cellulose nanofibers also allows them to disperse otherwise hydrophobic materials like carbon nanotubes very efficiently in water. As a result, nanocelluloses have been used for bottom‐up assembly of a plethora of smart, electronic nanocomposites in areas ranging from energy and sensors to robotics.^[^ ^24^ ^]^

**Table 1:** The most interesting properties of cellulose basic materials and nanomaterials.

Cellulose Based Materials	Important metrics
Paper	Mechanical: Tensile strength 100 MPa Mechanical: Youngs Modulus 4 GPa Dielectric constant (dry paper) 4^[^ ^28^ ^]^ Thermal conductivity 0.15 Wm^−1^K^−1^ Specific heat 1.5 kJ kg^−1^K^−1^ Production scale >100 million tonnes/year 2020^[^ ^6^ ^]^
Wood	Maximum Strength >40 MPa Production scale of sawn wood products >500 million tonnes/year 2020^[^ ^6^ ^]^
Regenerated cellulose	Dielectric loss (dry) <0.1^[^ ^28^ ^]^ Highest electrical breakdown strength (dry) 500 mV m^−1[^ ^29^ ^]^ Insoluble in water and most organic solvents
**Cellulose nanomaterials**	
Single cellulose nanofiber	Stiffness of up to 140–150 GPa^[^ ^19^ ^]^ Tensile strength 5–10 GPa^[^ ^21^ ^]^ Highest aspect ratio >1000
Cellulose nanofiber structural materials (microfibers, sheets, hydrogels, foams)	Highest Surface area (aerogels) 500 m^2^ g^−1[^ ^23^ ^]^ Lowest weight (aerogels) 0.001 g cm^−3[^ ^23^ ^]^ Lowest thermal conductivity (aerogels) 0.02 Wm^−1^K^−1[^ ^23^ ^]^ Highest water uptake (selfsuporting hydrogels) 99.99%^[^ ^22^ ^]^ Highest tensile strength >1.5 GPa (aligned nanofibers)^[^ ^30^ ^]^ Highest Young's modulus >80 GPa (aligned nanofibers)^[^ ^30^ ^]^ Low thermal expansion (foams) 10^−6^ K^−1[^ ^28^ ^]^
Nanostructured wood	Highest tensile strength (compressed wood) 600 MPa^[^ ^26^ ^]^ Lowest Thermal conductivity 0.03 W mK^−1[^ ^31^ ^]^ Highest ion conduction 0.2 S cm^−1[^ ^32^ ^]^

While nanocellulose is used in the bottom‐up assembly of new materials, an alternative approach has been to utilize and partly keep the nanostructure in the wood, in which the nanocellulose and its higher‐order structures largely define material properties. Already in the 1990s, the idea of transparent wood was demonstrated by removing and bleaching colored compounds in wood, such as lignin, and filling the wood structure with a matrix of matching refractive index.^[^ ^25^ ^]^ This approach was later used to create new types of wood‐based construction materials, which enhanced the strength and other properties of native wood, which will be discussed in the next chapter on construction materials.^[^ ^26^ ^]^ These ideas have opened up a new branch in cellulose‐based materials called “wood‐ nanotechnology.”^[^ ^27^ ^]^ This field has the potential to re‐engineer wood to a more advanced construction material in the future, as will be discussed in detail in the next chapter.

Table 1 lists the interesting properties of the major classes of basic and advanced nanomaterials currently created from cellulose. As wood and cellulose now enter the nano‐age, the list expands into fundamental properties of nanocellulose and some of its offspring materials that we are now exploiting in bottom‐up and top‐down self‐assembly approaches, although we are still far from having discovered or fulfilled the full potential of cellulose nanotechnology.

In the following sections of this perspective, we first focus on two areas where we foresee that the use of cellulose‐based advanced materials could lead to very large‐scale change toward a more sustainable society. We then highlight some of the current obstacles that need to be solved and provide perspective for the future areas where materials science should focus on enabling the revolutionary cellulose‐based materials of the future.

## 5 Two Near‐Term Possibilities with Great Potential

### 5.1 Cellulose‐Based Electronic Substrates

The modern transformation of society was largely brought by the rapid development of electronics in the past decades, and there are no signs that the development of electronics is slowing down. On the contrary, the development of society and technology and emerging concepts such as the Internet of Things (IoT), make electronic devices and functions ever more ubiquitous. As with all technology transformations, electronics come with an environmental footprint, and a large share of this footprint originates from the materials in the substate onto which electronic circuits are built, the so‐called printed circuit boards (PCBs). Electronic waste reached more than 50 million tonnes^[^ ^33^ ^]^ globally in 2019, with a low recycling rate. The PCB substrates constitute a significant component of this waste. Most of our electronics are fabricated using rigid PCBs based on flame retarding glass fiber‐reinforced composites and partly on flexible PCB substrates of fossil‐based polyimide. These nonrenewable materials are expensive and have substantial environmental impacts. A large part of the environmental impact of today's PCBs stems from the commonly used subtractive manufacturing of conducting traces involving etching processes^[^ ^34^ ^]^ and the high environmental costs of separating and recovering metals.^[^ ^35^ ^]^ Reducing the environmental footprint of electronics using PCBs built from renewable and recyclable materials is paramount to a sustainable future and digital and green transitions. The prospect of reintroducing cellulose into electronic substrates is attractive, and the challenges are briefly discussed here.

One can first note that early PCBs were indeed composites of cotton‐reinforced phenol resins. These early cellulose‐containing PCBs were gradually replaced by flame‐retardant glass fiber‐reinforced epoxy resins. The re‐introduction of cellulose in PCBs for advanced circuits is challenged by the many demanding requirements placed on the material, such as its operational conditions and manufacturing processes of PCBs and circuits.

PCB devices are multi‐material and multi‐component structures, and most PCBs are constructed as multilayer structures. This places high demands on matching dimensional expansion in response to variations in temperature and moisture level. Depending on application requirements, dielectric properties, flame retardancy, and tolerance to process and operation conditions are required for manufacturing processes and operations. Under dry conditions, cellulose is dimensionally stable with thermal expansion as low as 10^−6^ K^−1^ and a dielectric loss factor of 0.1 (Table 1), which is adequate for a PCB material for most low‐frequency applications. The main hindrance to a broader implementation of cellulosic fibers in PCBs is that these properties depend strongly on the moisture content and the hygroscopic nature of cellulose. The dielectric loss factor, in particular, depends strongly on the moisture content.^[^ ^36^ ^]^

Approaches to reduce the water uptake include chemical modifications or composites.^[^ ^37^ ^]^ Another strategy can be to encapsulate cellulose in matrixes deposited from waterborne solutions to form a highly uniform coating at the finest nano levels. These coatings could keep mechanical integrity and enable adaptable hybrid material. Similar strategies have recently been shown for glass‐coated metal fiber composites.^[^ ^38^ ^]^

Another approach is to stabilize the fiber structure using a matrix polymer. Products like Soluboards marketed by JIVA materials (Figure 3A) are cellulosic fibers in a matrix of polyvinyl alcohol. This composite material tolerates exposure to PCB processing conditions while disintegrating upon immersion in water for extended times.^[^ ^38^ ^]^ Cellulose papers can also be stabilized to hygroexpansion structurally by laminating cellulose paper onto glass fiber webs, which is the case in so‐called CEM‐1 boards (Figure 3B) from the company Ansys. In this composite board, the hygroexpansion in the plane of the board is limited by the glass fiber structure. For single‐layer PCBs where the circuitry is placed on only one layer, CEM‐1 boards are sufficient if the requirements for flame retardancy are low. Most advanced circuits are built on multilayer PCBs, structures where conducting traces are placed on many layers to provide the routing needed for the circuit.

In multilayer PCBs, routing between conducting traces at different layers is provided by vertical interconnect access (via) channels. These are typically constructed by metal plating on the inside of a drilled hole. A common failure mode of PCBs is the rupture of via channels due to expansion in the z‐direction of a PCB. Via failure due to hygroexpansion in the z‐direction is a limiting factor for cellulose‐based multilayer PCBs. A case in point is provided by the CEM‐1 boards. As the cellulose fibers in the sheet are mainly oriented in the plane, there is little restriction to expansion in the thickness direction of the paper sheet. This makes routing between layers through vias difficult. As a consequence, such substrates are practically limited to single‐layer structures. Broader applicability of cellulose‐based circuit board structures in multilayer PCBs would demand better methods to limit hygroexpansion in the direction out of the plane (z‐direction). Although this may be possible, reducing the interactions of the cellulose with water too much may render the composite intractable to common recycling processes that are desired for facile recyclability.

To realize recyclable PCBs that can tolerate PCB processing and operation, with a minimum of z‐direction expansion, while being recyclable with existing methods, there is a need to control the interactions between cellulose and water. Until this can be realized, the applicability of cellulosic composites in PCB is limited to single‐ and double‐layer structures.

Fire retardancy is another requirement in most PCB devices carrying currents that need to be met by cellulosic materials. Current substrates must conform to fire standards like the UL94‐V0, which requires that burning should stop within 10 s. There are methods to impart fire retardancy to cellulose fibers, like chemical modification of cellulose surface, e.g., using phosphorylation chemistry,^[^ ^39^ ^]^ or methods based on embedding cellulose fibers in inorganic SiOx that may contain heat inhibiting functionalities preventing oxygen initiation and favoring smoldering behaviors.^[^ ^40^ ^]^

Until these methods are industrialized, however, cellulose boards are practically limited to applications with single‐layer PCBs carrying weak currents and not operating at very high frequencies, such as in “Internet of Thing” (IoT) devices. This limited market segment is expected to grow as IoT devices are expected to become ubiquitous. A wider implementation of cellulose‐based PCB materials calls for new developments for controlling hygroexpansion and flame retardancy.

### 5.2 Future Construction: Cellulose‐Based Materials Replacing Steel and Cement

Construction materials for tall buildings of steel began use in the USA in the mid‐19th century, and decades later, concrete started being used in high‐rises. Steel and cement are the bulk, high‐strength materials used in all major constructions today. The production of cement leads to an annual emission of 2.3 Gt of carbon dioxide^[^ ^41^ ^]^ (6.5% of total emissions), and steel stands for 2.6 Gt of carbon emission into the atmosphere (7% of total), which is larger than the emission of all planes, ships, and cars combined. Replacing even a fraction of these materials with sustainable alternatives makes important contributions toward a sustainable future civilization.

Wood‐based construction materials are probably our only alternative. Today, the most widely used cellulose‐based construction material is mass timber. The two main mass timber products are cross‐laminated timber (CLT), made from strips of timber that are layered and glued perpendicularly under pressure to form a strong singular piece, and glue laminated timber (glulam), which has its layers glued together in parallel rather than perpendicular. CLT is stable and can be used as load‐bearing walls and floors.^[^ ^42^ ^]^ Glulam is much more suited to linear building elements such as columns and beams than structural walls. These columns and beams form framed systems but utilize non‐structural materials to fill the areas between the structural supports forming walls.

**Figure 4:**
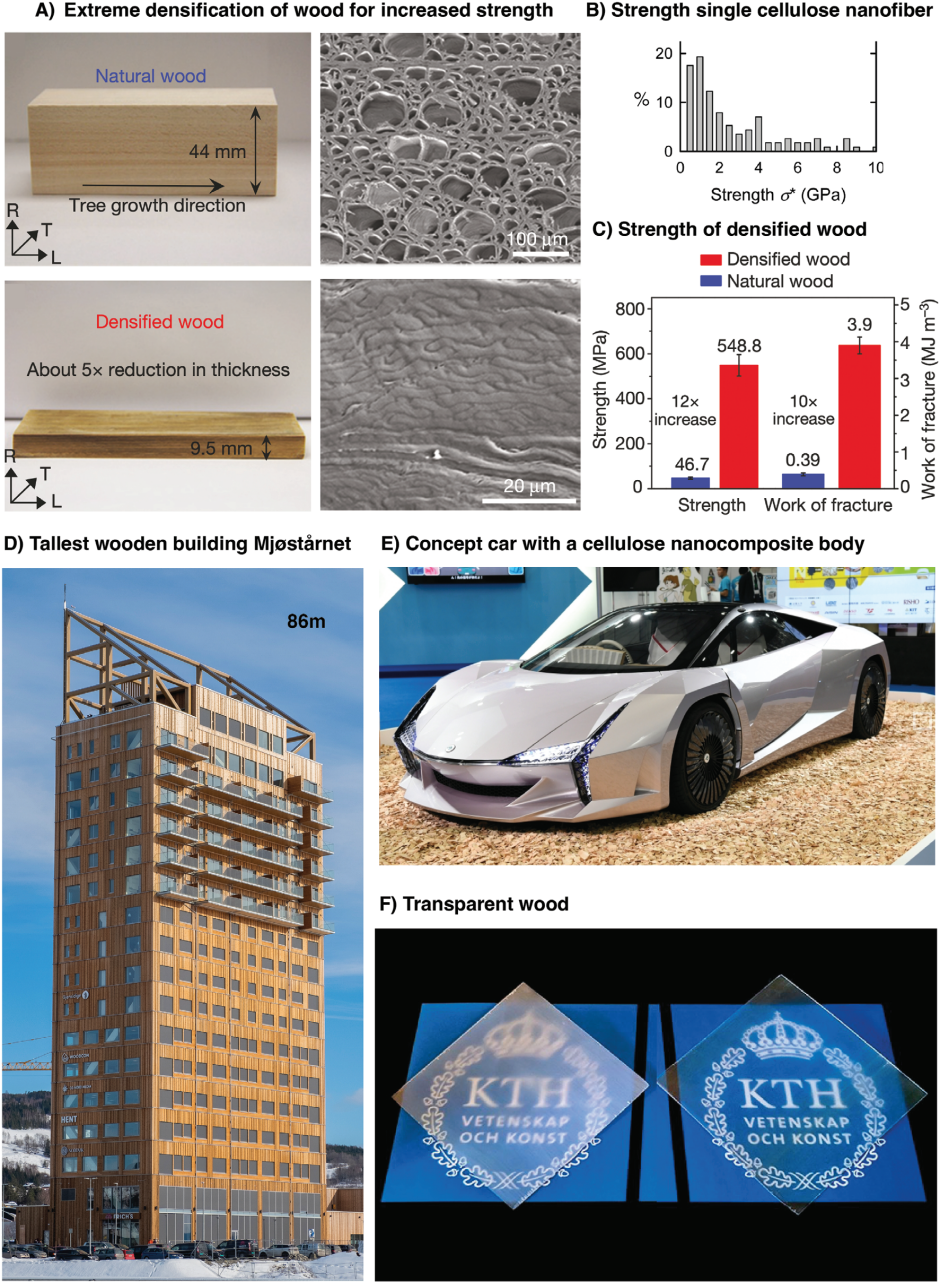
Wood as a construction material: A) Microstructure of natural wood versus densified wood. Reproduced from Ref. [26] with permission from Nature Publishing group. B) Measures of the strength of single cellulose nanofibers. Reproduced from Ref. [21] with permission from Biomacromolecules. C) The measured strength of densified wood versus natural wood with over 10× increase.^[^ ^26^ ^]^ D) Photo of the world's tallest wooden building to date situated in Norway (Reproduced with permission Moelven). E) Photo of a concept car with a body and parts made from nanocellulose composite materials (provided by the Ministry of the Environment, Japan). F) Photo of two transparent woods with different haze (with permission from Céline Montanari).

For steel and cement, there are many tested methods for predicting horizontal stiffness with calculation models usually calibrated through scientific experiments. Corresponding construction systems for wood have only been around for a few decades, which means that engineering experience, particularly regarding stabilization, is limited. We only have limited experience with tall wooden buildings, particularly those with more than eight or nine floors. Examples of taller budlings do, however, exist. At the time of its completion in 2019, the 86‐meter tall “Mjøstårnet” tower in Norway (**Figure** **4**D) is fabricated with glulam columns and elevator shafts made entirely from CLT. Mass timber wood has a very high strength‐to‐weight ratio, making it beneficial also for mass fabrication and transportation. It also has high thermal mass for insulation and fire resistance as it forms a char‐protecting layer that prevents fire. The only physical shortcoming of the current mass timber is the tensile strength and axial stiffness. Glulam, for example, has much lower strength and stiffness than regular structural steel S355, named based on its minimum yield strength of 355 MPa, but can reach a tensile strength of 680 MPa. The tensile strength of standard glulam is ≈1/10 of that of steel. As a result, the stabilization system in wood for tall buildings, where the requirement for horizontal stiffness is critical, can lead to very large cross‐sectional dimensions that, in many cases, may be impractical.

To compare this with wood, we could consider the nanomechanical measurement of the smallest building block of wood: the cellulose nanofiber. Iwamato measured the elastic modulus of wood to as high as 200 GPa^[^ ^19^ ^]^ and we measured in 2013 the tensile strength of carboxyl‐functionalized CNFs to average values ≈6 GPa and highest values astonishingly surpassing 10 GPa^[^ ^21^ ^]^ (Figure 4B) These are important fundamental metrics as they are indicative of the future potential for building ever stronger materials with cellulose using clever engineering. Similar trends have indeed been seen in other fields of nanotechnology, for example with carbon nanotubes. Indeed, the tensile strength of carbon nanotube composites has increased by over 20% per year.^[^ ^43^ ^]^ For CNT composites, two combined developments have pushed this development: i) improved quality and consistency of the basic nanomaterial,^[^ ^44^ ^]^ ii) technical development allowing alignment and ordering of the material. Similar strategies have recently been pursued for CNFs where refinement nanomaterial production combined with intelligent methods to align these nanofibers have resulted in pure cellulosic structures that now surpass 1 GPa in tensile strength.^[^ ^30^, ^45^ ^]^ We are, therefore, already achieving cellulose‐based materials stronger than commercial structural steel thanks to the inherent mechanical nature of the nanocellulose fibers.

These bottom‐up production methods from nanocellulose cannot, however, be scaled up to achieve materials for construction. Instead, we need to turn toward top‐down cellulose nanotechnology. One approach that has been pursued since the early 1900s is timber densification.^[^ ^46^ ^]^ Here, the cellular structure of timber is modified through compression and chemical modification to enhance its properties, such as strength and hardness. A recent leap in this direction^[^ ^26^ ^]^ has shown that wood can be treated in a boiling process in NaOH and Na_2_SO_3_ to partially remove lignin and hemicellulose from the natural wood, which is then compressed and densified (Figure 4A). In this process, the cell walls in the densified wood at the microscale end up in a higher degree of alignment with their cellulose nanofiber constituents, which now bind more tightly. Consequently, the energy needed to fracture densified wood becomes much higher. The densification of wood also greatly reduces both the quantity and size of defects found in vessels to tracheids and leads to one order of magnitude higher strength than that of natural wood with strength strengths over 500 MPa at high work of fracture. Densified stronger wood is still not produced at scale, but startup companies are pursuing future mass production with products like MettleWood.

Densification of wood is most probably going to find its most extensive use for structural elements. Still, the principle of densification also provides opportunities such as making wood transparent^[^ ^25^ ^]^ to even partly replace glass structures with wood‐based materials.^[^ ^47^ ^]^ Cellulose nanomaterials have also been used as a lightweight and sustainable material to replace parts in cars. The Ministry of the Environment of Japan has launched a concept car called Nano Cellulose Vehicle NVC, aiming to reduce the total weight of a vehicle by 10% (Figure 4E).^[^ ^48^ ^]^

While the pursuit of greener steel and cement is an important step for future sustainable constructions, this section highlights that cellulose‐based materials possess all the necessary properties to replace the bulk of structural steel and cement in massive constructions in the future. This is enabled using state‐of‐the‐art materials like glulam and CLT complemented by super strong compressed wood and combined with new computer‐based architectural design tools. Future cities could host skyscrapers surpassing 100 m, which not only lead to a decrease in carbon dioxide emissions but also act as carbon sinks.

## 6 The Role of Water is the Key that Unlocks the Door to the Future

**Figure 5:**
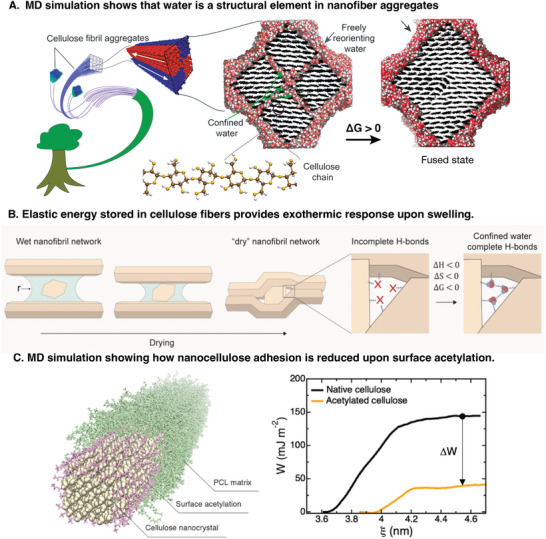
A) Water confined within cellulose fibril aggregates was shown to be thermodynamically stable, thus contributing to their stability. Adapted under the terms of the CC‐BY‐4.0 license.^[^ ^53^ ^]^ Copyright 2022, The authors. B) Drying of nano fibril networks induces deformation of the fibrils. The stored elastic energy is released upon swelling, giving an exothermic response. Due to packing restraints, there are nano‐sized pores where water molecules can reside even in “dry" conditions. Reproduced under the terms of the CC‐BY‐4.0 license.^[^ ^52^ ^]^ Copyright 2022, The authors. C) Simulations show how surface acetylation significantly reduces the adhesion between cellulose nanoparticles in a PCL matrix, leading to better dispersion and less aggregation in nanocomposites. Adapted under the terms of the CC‐BY‐4.0 license.^[^ ^56^ ^]^ Copyright 2023, The authors.

As discussed throughout the article, cellulose self‐assembles in water, forming hierarchical structures starting with the cellulose nanofibers and growing into millions of times larger structures in the form of trees and plants. Interaction with water is, therefore, a critical property of cellulosic materials. A technical challenge for some applications is that cellulose‐based materials swell in contact with moisture (e.g., for circuit boards as described above), both in the form of vapor and liquid water. Paradoxically, the cellulose crystal is impenetrable to water,^[^ ^49^ ^]^ and the cellulose polymer is completely insoluble in water despite its hydrophilicity. This means that the only place water can go is between the crystalline domains. Swelling is often studied using moisture sorption isotherms. Swelling of polysaccharides, including cellulose, is generally exothermic. This has led to many authors using BET formalism to explain moisture sorption isotherms with the assumption that swelling is due to surface adsorption, driven by the formation of water‐carbohydrate hydrogen bonds. However, as pointed out by Nishiyama,^[^ ^50^ ^]^ the BET surface area that comes out of such analysis is typically much lower than the area measured by other means, e.g., nitrogen gas sorption. This, he argues, shows that polysaccharide swelling is not a surface phenomenon driven by hydrogen bond formation. Indeed, in the compact dry state, there are very few unsaturated hydrogen bonds, as the carbohydrates are prone to form hydrogen bonds among themselves. Thus, the formation of new water‐carbohydrate hydrogen bonds is accompanied by the destruction of carbohydrate‐carbohydrate bonds, leading to a net energy gain close to zero. Instead, the exothermic response is driven by the release of elastic energy as stiff polysaccharides relax from strained conformations in the dry state (**Figure** **5**B).

Due to the abundance of polar groups in cellulose, hydrogen bonds are commonly used to explain its desirable properties, including high stiffness and strength. Such an explanation is, however, overly simplified, as entropy is the main driving force for polymer association in water^[^ ^51^ ^]^ and cellulose in particular.^[^ ^52^ ^]^ The cellulose chain is, in fact, an amphiphilic molecule^51^ and this is reflected in cellulose materials. A clear everyday example is that ordinary tissue paper readily absorbs both water and oil. The standard method to quantify hydrophilicity is through contact angle measurements. Indeed, water contact angles on non‐modified cellulose or wood substrates are finite, and their magnitudes are below 90°, i.e., in the hydrophilic regime. In combination with an unusually high surface tension compared to other liquids, water's adhesion to cellulose is higher than most organic liquids.^[^ ^52^ ^]^ Consequently, the water present at the cellulose surfaces is very hard to remove, and even “dry” cellulose materials typically contain up to ≈10% water. Additionally, even the most compact cellulose structures, like nanopaper made from cellulose nanofibers or compressed wood, will have nanoscale pores and imperfections at the interface between fibrils where water molecules can reside (Figure 5A,B). Molecular dynamics simulations could later confirm that such confined water was thermodynamically stable as opposed to just kinetically trapped^[^ ^53^ ^]^ (Figure 5A). In other words, water is an intrinsic component of cellulose materials, and we should learn to take advantage of the special relationship between the 2.^[^ ^54^ ^]^ Indeed, living trees have ≈20 wt.% water in their secondary cell walls, yet they can grow more than 100 m tall and become several thousands of years old. Unlike synthetic polymers, water can be beneficial for the mechanical properties of fibers, improving both mechanical strength and toughness. Surface confined water is also responsible for the remarkable efficiency of chemical reactions in cellulose materials.^[^ ^55^ ^]^

The recent combined advancements in cellulose nanotechnology and the ability to simulate more extensive macromolecular systems are opening new paths for the study of cellulose‐water interactions. One example relates to the contact angle measurements mentioned above. At the experimental scale, measurements are complicated by significant effects from surface roughness and porosity. Here, simulated atomistic‐scale contact angles complement experimental results. Although suffering from less‐than‐perfect empirical interaction potentials, simulated contact angles are closer to the “ideal” wettability due to the use of extended and perfectly smooth substrates. These combined experimental and MD simulations are an exciting path forward for gaining insight into optimizing the parameter sets of models to describe cellulose‐based macromolecular systems.

Future studies using refined and more extensive MD simulations will lead to better models with higher predictive power also for material properties. Perhaps most importantly, we could use models to search through a space of chemical possibilities where different functional groups on the cellulose molecule and/or novel polymers and nanomaterials could be discovered toward advanced future nanocomposites. Works in that direction include the effect of surface acetylation on CNC aggregation in suspension and in polymer matrices^[^ ^56^ ^]^ (Figure 5C) and on stress transfer between cellulose nanoparticles.^[^ ^57^ ^]^ We end this perspective by discussing some more revolutionary aspects that can be enabled by these emerging materials.

## 7 Revolutionary Future Materials

### 7.1 High Surface Area and Interface with Water Enable New Functions

The hierarchical cellulose structure at the fiber‐to‐wood level already provides a large surface area, making it a versatile material for many applications. With the advent of cellulose nanotechnology, bottom‐up assembly from native cellulose nanofibers has opened the path for the fabrication of aerogels with even higher porosity and surface areas surpassing 500 m^2^ g^−1^ (Table 1). These materials, based on pristine cellulose, have shown promise in air pollutant removal or as space dust collector due to their expansive surface area and the ability to host nanoparticles, or in energy and environmental applications such as collecting water from the atmosphere or industrial mining and communal wastewater treatment.^[^ ^58^ ^]^

Many possibilities for chemical surface functionalization of the cellulose molecule have been explored over the last few decades, mainly using organic surface modifications, e.g., esterification or etherification, at the Ångström to the nanometric range. Organic surface modification affects the outer surface at distances between 1–10 nm, whereas polymer grafting can extend the modification up to 50 nm, particularly when grafted polymers form brush‐like structures that protrude from the cellulose surface.^[^ ^59^ ^]^ In structured approaches like layer‐by‐layer assembly, the build‐up of many layers can extend the dimension of the modified surface to beyond 50 nm.^[^ ^60^ ^]^

The native cellulose material surfaces can, however, not only be functionalized organically, which primarily is modifications directed toward the interface function of the cellulose encompassing solubility and compatibility of the already hierarchal built cellulose nanofibrils. Cellulose can also be modified inorganically to enhance properties like electric and thermal conductivity,^[^ ^61^ ^]^ catalysis,^[^ ^62^ ^]^ ion exchange, and adsorption of pollutants^[^ ^63^ ^]^ toward applications such as batteries, supercapacitors, and environmental remediation. Since cellulose and its nanofibers are among the most stable biopolymers, being stable in harsh pH conditions also at high temperatures, it is possible to modify the surface area of advanced hierarchical structures of cellulose using inorganic precursors due to the inherent crystalline nature of the fibrils. In this context, only a few competitive materials exist with the same functionality at much smaller representation or limited accessibility in nature, which are the cousins of cellulose: chitin or chitosan.^[^ ^3^ ^]^

It is suggested that the area of these inorganic‐organic cellulose hybrid materials holds great promise in directed condensation strategies wherein multiple or combined inorganic assemblies can be envisioned. The condensation chemistry approach, in association with cellulose nanofibers allows for true hybrid materials to be explored in different applications. One such example is the incorporation of cellulose nanofibers into metal‐organic frameworks (MOFs). In these hybrids, cellulose provides the structural backbone that enhances mechanical stability, while the active site MOFs enhance the material's ability to capture pollutants or facilitate catalytic reactions.^[^ ^64^ ^]^ Other examples involve the grafting of inorganic nanoparticles to the frameworks of cellulose nanofibers. The role of cellulose is here to promote growth and organize the structure of the inorganic materials to provide functionalities ranging from antibacterial silver particles toward water treatments in filters^[^ ^65^ ^]^ to magnetic functions providing unique and strong absorption of pollutants such as arsenic in the groundwater or electronic sensing.^[^ ^66^ ^]^

**Figure 6:**
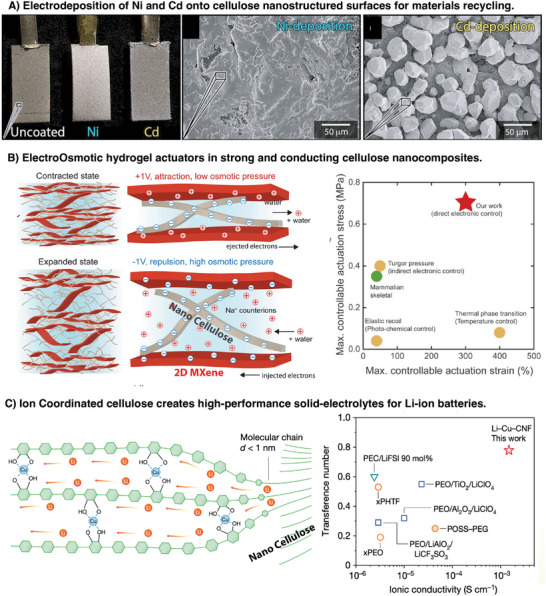
Toward revolutionary materials: A) Photograph of Ni and Cd coated cellulose substrates and corresponding close‐up SEMs. Reproduced from Ref. [67] with permission from the Royal Society of Chemistry. B) Electroosmotic (ECO) hydrogel actuator fabricated from cellulose nanofibers composited with electronically conductive CNTs or 2D MXenes. Reproduced from Ref. [69, 70] under the terms of the CC‐BY‐4.0 license. Copyright 2024, The authors. C) Cu^2+^ ion coordination opens cellulose chains, enabling better conduction in solid Li‐ion batteries. Reproduced from Ref. [71] with permission from Nature Publishing Group.

Another underutilized area for future use of cellulose is their use as biobased electroactive additives for industrial processes. The cellulose nanomaterials could challenge the nature of classical electrodeposition on the finest scales since it effectively enables control of electrodeposition rates by changing the structure and dynamics of reaction in water at the surface. This point has, for example, been demonstrated in the hierarchical growth of dendrimer metal crystals that otherwise would not form in the absence of cellulose crystals.^[^ ^67^ ^]^ This phenomenon has been demonstrated during depositions from mixed Ni/Cd metal solutions to increase the deposition rate of Cd, highlighting the great potential for recycling materials (**Figure** **6**A). We hypothesize that the water in arrested states created by colloidal gels/glasses of cellulose nanofibers in more or less concentrated states^[^ ^68^ ^]^ changes the nature of inorganic material growth, providing new possibilities for industrial processing at scale in water. The dynamic and structural nature of the water in colloidal glasses/gel is still not fully understood, and as noted in the previous sections further simulations and experiments are the key to unlocking this new potential.

### 7.2 Cellulose Nano‐Age Converges with the Electronic Nano‐Age

The proliferation of nanomaterials from conducting polymers^[^ ^72^ ^]^ (e.g., PEDOT:PSS) to carbon nanotubes CNTs,^[^ ^73^ ^]^ to 2D materials (e.g., graphene and beyond like 2D MXenes,^[^ ^74^ ^]^ and 2D TMDs)^[^ ^75^ ^]^ has happened in parallel to the development of cellulose nanotechnology. A plethora of possibilities is, therefore, now enabled by combining cellulose nanomaterials with electronic nanomaterials. The most apparent materials are the ones that combine the essential properties of the two, such as the mechanical strength of the nanocellulose combined with the electronic conductivity of MXene.^[^ ^61^ ^]^

Another direction revolves around using cellulose nanomaterials, like nanopapers, as a substrate for the monolithic integration of electronics,^[^ ^76^, ^77^ ^]^ or as mixed water dispersions of cellulose nanofibers with water‐dispersible conducting nanomaterials to achieve electronic composites.^[^ ^61^ ^]^ Such materials are, of course, closer to industrialization, and as argued above on the subject of PCBs, it is indeed suggested that new substrates for electronics can have a considerable impact.

However, using cellulose as a mere substrate or structural element in a nanocomposite is not revolutionary. The novel cellulose‐based materials of the future must rely on the complex nanostructures that form in the bulk of the material, self‐assembled from cellulose and synthetic functional nanomaterials. The function of these composite materials emerges from the complex interplay between electronics, ions, water, and the nanostructure of the electronic composite. A small step in this direction utilizes the amphiphilic nature of cellulose nanofibers (or polymer derivatives like CMC), utilizing this molecule as a dispersing agent. We and others have shown that cellulose nanofibers are excellent dispersing agents for CNTs,^[^ ^78^, ^79^ ^]^ and 2D materials like MoS_2_ or boron nitride,^[^ ^80^ ^]^ which are hardly dispersible in water by themselves. Here cellulose has a dual role in enhancing and providing colloidal properties to electronic nanomaterials and then be used toward composites.

However, very few advanced emergent functions have been shown that rely on and draw their function from the complex interplay between electrons, ions, water, and the nanostructures. We highlight here two interesting examples:

One includes the creation of nanochannels in which the shape of the conduit channels gives rise to enhanced ion conduction for batteries.^[^ ^71^ ^]^ This happens through the coordination of Cu^2+^ ions, which open the channels (Figure 6C) within the cellulose nanofibers, creating 1D conduction nanochannels. It is hypothesized that the abundant oxygen‐containing groups of cellulose and the bound water assist the movement of Li+. This mechanism is decoupled from the segmental motion of the polymer leading to new highly enhanced conduction not captured by classical polymer physics, enabling a new generation of solid electrolyte Li‐ion batteries. It is astonishing that these Li‐ion batteries work exceptionally well because of the presence of water, which otherwise is deemed detrimental to Li‐ion batteries. As we argued in the previous section, bound water is hard to remove, so the water does not hydrolyze or degrade here. Again, the role and structure of water is the most important area that needs to be understood for such revolutionary future materials like these in energy storage.

The other example is an electroosmotic (ECO) hydrogel actuator fabricated from cellulose nanofibers composited with electronically conductive CNTs or 2D MXenes.^[^ ^69^, ^70^ ^]^ In these actuators, the capacitive double‐layer charge of nanocomposite material (Figure 6B) leads to a large change in the chemical charge of the bulk of the hydrogel. This, in turn, results in an increased swelling pressure through osmosis, where up to 700 water molecules per ion/electron pair dynamically pass in and out of the material. The in‐plane stiffness of the ECO hydrogel transduces the swelling pressure into expansion in the out‐of‐plane direction because of the anisotropic structure formed by the cellulose nanofibers. These actuators are stronger than human muscles and show many other interesting properties, like dynamic pore size control at the mesoscale.

These two examples demonstrate that to achieve truly revolutionary electronic materials, we should pursue two parallel developments: i) Further understand the complex interaction of water and its connection to the dynamics of electrons and ions as structures assemble into complex shapes and as these shapes impose new functions. ii) Design and fabricate future electronic nanomaterials that specifically interact with and inherit or interplay with the supramolecular assembly of the cellulose molecule. These materials would allow us to engineer structures beyond randomly ordered or semi‐ordered nanowire networks. We could, for example, design de‐novo conducting polymers with engineered backbones that, like cellulose, rely on the organizational role of hydrogen‐bonded networks for joint self‐assembly into higher‐order structures. Such synthetic electroactive polymers could enable truly bottom‐up engineered electronic nanocomposites with properties that arise from local mesoscopic order rather than macroscopic random networks or simple anisotropy, which defined the function in the two examples above. The most compelling functions of these future devices emanate from the complex interplay between electrons, ion: following the physics of mixed ionic electronic conductors^[^ ^81^, ^82^ ^]^ and their coupling to water through osmosis and other mechanisms.

Such materials would self‐assemble from water into well‐defined 3D nanostructure, and their function would be achieved at nanometre resolution inside the bulk of the material. This could, for example, include neuromorphic computers where 3D junctions would form dense 3D arrays of computational elements such as electrochemical RAMs,^[^ ^82^ ^]^ allowing a new generation of water‐based computers that would be more similar to our brain. Such computational systems could be integrated with energy storage, sensors, and actuators to form monolithic intelligent systems.

An even more revolutionary concept could use the yet poorly explored mechanism of transport (e.g., the “proposed radical electrons” in the cellulose itself.^[^ ^83^, ^84^ ^]^

Naturally, since cellulose is already a bio‐based and biocompatible material, we can also use structural materials like hydrogels for seeding or hosting living cells^[^ ^85^ ^]^ or as extracellular matrices for tissue engineering. This has already been shown for 3D printed tissue engineering,^[^ ^86^ ^]^ and there are commercialized products like “GrowDex” from UPM Biomedical. Advanced wood‐based biohybrids have also been shown, for example, by colonizing wood with fungus to achieve bioluminescence.^[^ ^87^ ^]^ The next generation of electronic cellulose nanocomposites combined with advanced tissue engineering could enable new electronic biohybrids.

## 8 Coda

Being by far the most abundant sustainable material on Earth, wood and its prominent constituent cellulose are among the most important sustainable materials we have at our disposal to solve major societal challenges. Although cellulose and wood are carbon‐neutral materials in a closed cycle, sustainable usage requires further developments in the wood industry; The pulp industry has come far in cleaning up the isolation processes, due to environmental regulations. we still need better processes to preserve biodiversity in forests or agricultural land. A partial solution is agricultural waste as a source of cellulose.^[^ ^88^ ^]^ In general, this grand challenge requires broad interdisciplinary research and development, which is partly impeded even by the structure of academia itself which should evolve to be less tribal‐like, and allow for more collaboration.^[^ ^89^ ^]^

We envision here and highlight directions toward next‐generation advanced cellulose‐based construction material, which can be used for massive buildings like skyscrapers and for moving toward renewable electronic substrates. These are two areas of immense scale and will be of seminal importance for our societies in the near future. In the long term, we highlight how research on cellulose nanotechnology – especially its connection and dynamics with water, combined with the advances in electronic nanomaterials and biotechnology will lead to revolutionary materials.

We only outline the possibilities with such materials which are currently beyond our imagination, but they can address critical societal issues in many areas from materials recycling to energy, intelligent systems, and biohybrids.

## Conflict of Interest

The authors declare no conflict of interest.
